# Proteomic analysis reveals the diversity and complexity of membrane proteins in chickpea (*Cicer arietinum* L.)

**DOI:** 10.1186/1477-5956-10-59

**Published:** 2012-10-02

**Authors:** Dinesh Kumar Jaiswal, Doel Ray, Pratigya Subba, Poonam Mishra, Saurabh Gayali, Asis Datta, Subhra Chakraborty, Niranjan Chakraborty

**Affiliations:** 1 National Institute of Plant Genome Research, JNU Campus, Aruna Asaf Ali Marg, New Delhi, 110067, India

**Keywords:** Grain legume, Membrane-associated proteins, 2-DE, Mass spectrometry, Transmembrane domain

## Abstract

**Background:**

Compartmentalization is a unique feature of eukaryotes that helps in maintaining cellular homeostasis not only in intra- and inter-organellar context, but also between the cells and the external environment. Plant cells are highly compartmentalized with a complex metabolic network governing various cellular events. The membranes are the most important constituents in such compartmentalization, and membrane-associated proteins play diverse roles in many cellular processes besides being part of integral component of many signaling cascades.

**Results:**

To obtain valuable insight into the dynamic repertoire of membrane proteins, we have developed a proteome reference map of a grain legume, chickpea, using two-dimensional gel electrophoresis. MALDI-TOF/TOF and LC-ESI-MS/MS analysis led to the identification of 91 proteins involved in a variety of cellular functions viz., bioenergy, stress-responsive and signal transduction, metabolism, protein synthesis and degradation, among others. Significantly, 70% of the identified proteins are putative integral membrane proteins, possessing transmembrane domains.

**Conclusions:**

The proteomic analysis revealed many resident integral membrane proteins as well as membrane-associated proteins including those not reported earlier. To our knowledge, this is the first report of membrane proteome from aerial tissues of a crop plant. The findings may provide a better understanding of the biochemical machinery of the plant membranes at the molecular level that might help in functional genomics studies of different developmental pathways and stress-responses.

## Background

Membranes are highly organized structures specially adapted to perform multiple functions in eukaryotic cells. They constitute the interface between the various cellular compartments and play a critical role in the exchange of substances and signals. Cell membranes consist of dynamic lipid-protein matrices wherein the lipid component provides a barrier to solute movement, and membrane-associated proteins perform unique biological roles in development as well as stress adaptation. The composition and dynamics of membrane proteins reflect their diverse function, and their nature and relative amount vary from one organellar membrane to another. The membranes associated with different organelles not only play important roles in maintaining the homeostasis within organelles, but also at the whole cell level. Approximately 30% of the cellular proteome is represented by membrane proteins [1]*.* These proteins perform some of the most important functions, including the regulation of cell signaling, cell-cell interactions, and intracellular compartmentalization [2]*.*

Due to the presence of highly specialized organelles such as plastids and vacuoles, the plant membrane proteome is more complex compared to that of animal cells. The pivotal role played by membrane proteins in many cellular processes makes their study imperative. However, the proteomic analysis of membrane proteins has always been hampered due to their recalcitrance to standard methodologies [3]. Indeed, membrane proteins are under-represented in large-scale proteomics and are challenging to work with. The proteomic analysis of membrane proteins has been impeded due to their hydrophobic nature, lesser abundance and physicochemical heterogeneity [4]. While much progress has been made in animal membrane proteomics, far fewer attempts have been made to characterize the plant membrane proteome [5-8].

Legumes are valuable agricultural crops that serve as important nutrient sources for human diet and animal feed worldwide. They serve as an important protein-rich food and have increasingly become a commercial commodity. The members of this family have unique features such as biological nitrogen fixation and symbioses with mycorrhizal fungi [9], which make them good experimental models for various studies. Chickpea is the most important grain legume and ranks third in terms of total global production [10,11]. The world’s total production of chickpea hovers around 8.5 million metric tons annually and is grown over 10 million hectares of land. Despite the importance of chickpea and its role in nutrition requirement in humans, it has remained outside the realm of large-scale functional genomics studies. Although in recent years, much attention has been given to chickpea genomics [12-16], there is still little information on its protein complement. In previous proteomic studies, we had developed an extracellular matrix- and nucleus-specific proteome of chickpea [17,18]. We report here the development of membrane proteome with an aim to use the reference map for more comprehensive characterization of the regulation and function of membrane proteins. Over 300 proteins were resolved using two-dimensional gel electrophoresis (2-DE), and MALDI-TOF/TOF and LC-ESI-MS/MS techniques were used to identify the proteins. The identified proteins were classified on the basis of their putative functions. Membrane-association of the identified proteins was validated by assessing their hydropathicity and the presence of transmembrane domains (TMDs). It is notable that ~70% of the dataset were predicted to be integral membrane proteins, which is considerably higher than other gel-based membrane proteomic analyses thus far. We have identified many candidates that are integral membrane proteins as well as membrane-associated proteins including those not reported earlier. This study may facilitate comparative proteomics as well as functional genomics studies of plant development and adaptation to various stresses.

## Results and discussion

### Isolation of membrane proteins

**Figure 1:**
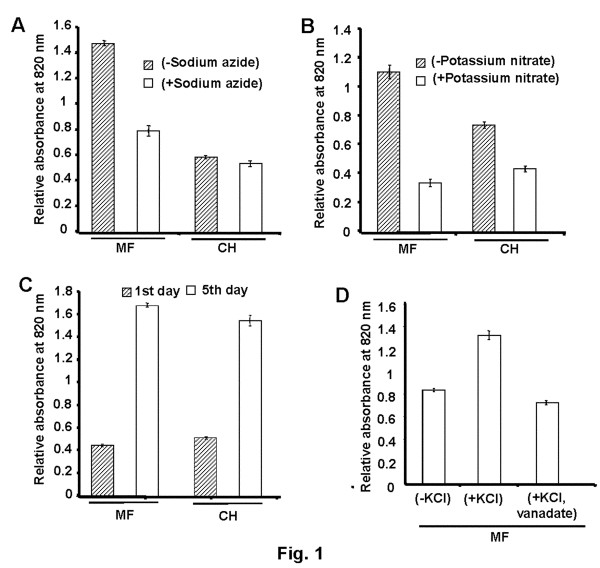
** Enzymatic Characterization of the Membrane Fraction.** The activities of marker enzymes associated with different organellar membranes were determined by spectrophotometric method. (**A**) Sodium azide-sensitive ATPase (mitochondrial membrane), (**B**) Potassium nitrate-sensitive ATPase (tonoplast), (**C**) Latent IDPase (Golgi membrane), and (**D**) K^+^-stimulated and sodium orthovanadate sensitive ATPase (plasma membrane). MF represents the membrane fraction, while CH represents the crude homogenate. All experiments were carried out in triplicates with at least two biological samples and average mean values were plotted against individual fractions.

The fractionation and selective enrichment of the microsomal fraction of chickpea was accomplished by differential centrifugation. It was systematically assessed for the enrichment of various subcellular membranous components using standard marker enzyme assays viz., vanadate-, azide-, nitrate-sensitive ATPase and latent IDPase for plasma membrane, mitochondrial membrane, tonoplast and Golgi membranes, respectively. The relative change in percent inhibition of ATPase activity associated with mitochondria and tonoplast was 2.29 and 1.68 fold, respectively in microsomal fraction compared to the crude homogenate (Figure 1A and B). Further, increase in relative fold-change in percent activity of latent IDPase in microsomal fraction was ~1.5 (Figure 1C). For plasma membrane ATPase, K^+^-stimulated increase in the percent activity was 1.68 fold, while vanadate-sensitive inhibition was 1.72 fold (Figure 1D). The activity of vanadate-sensitive ATPase could not be compared with crude homogenate as it had pH 8.0 and the assay was performed at pH 6.5. The higher level of activities of the marker enzymes in the membrane fraction indicates the enrichment of various subcellular membranous components.

**Figure 2:**
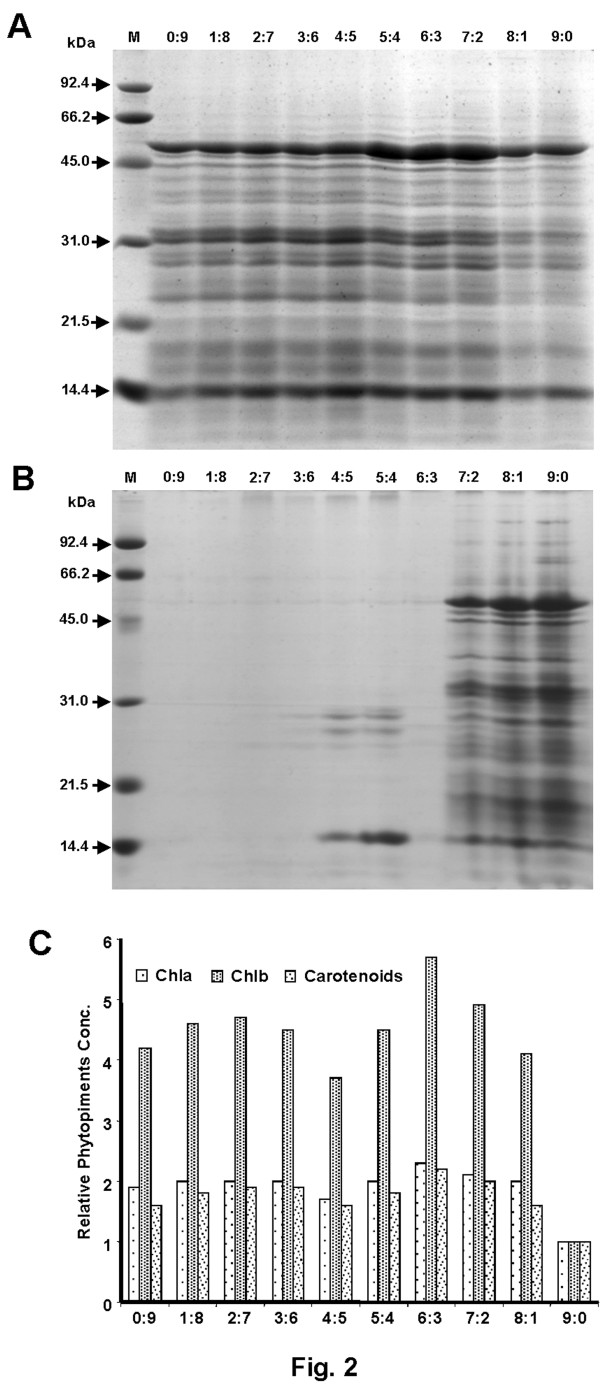
** Analysis of 1-D SDS-PAGE of the membrane proteins extracted using organic solvent procedure.** Varying ratios of chloroform/methanol (0:9 to 9:0) were used and respective fractions were resolved in 12.5% polyacrylamide gel followed by CBB R-250 staining. M indicates the molecular weight markers. Shown are (**A**) the profile of insoluble proteins, (**B**) proteins soluble in organic phase, and (**C**) various phytopigments viz., Chla, Chlb and carotenoids in the corresponding organic phase of various ratios of chloroform/methanol. Each experiment was performed in triplicates with at least two biological samples.

Major bottlenecks of membrane proteomics are the poor solubility and low abundance of the proteins, which limit their detection and identification. In this study, we focused on identification of the whole set of membrane proteins, including integral membrane proteins as well as peripheral membrane-associated proteins. It is understood that due to their physicochemical heterogeneity, there is no one-fit-all protocol for the extraction, solubilization, and separation of membrane proteins [19]. In order to achieve maximum representation of membrane proteins in one extraction procedure, we extracted the proteins using various ratios of chloroform/methanol. The proteins extracted from the insoluble pellet and soluble fraction (after acetone precipitation) corresponding to each chloroform/methanol ratio were then subjected to 1-D SDS-PAGE. While the insoluble proteins obtained from each chloroform/methanol extraction showed similar profile (Figure 2A), the proteins precipitated from their corresponding organic phase showed differential profile (Figure 2B). The presence of phytopigments viz., chlorophyll-a, b and carotenoids was also examined. The insoluble proteins obtained from 6:3 ratio of organic mixture was least green when compared to the others, which is corroborated by the content of contaminating phytopigments being maximal in its organic phase (Figure 2C). Taken together, these results suggest that 6:3 ratio of chloroform/methanol is optimal considering the maximal extraction of membrane proteins with least phytopigments contamination. The proteins from the corresponding pellet fraction were used to perform 2-DE. 

### Development of membrane proteome

**Figure 3:**
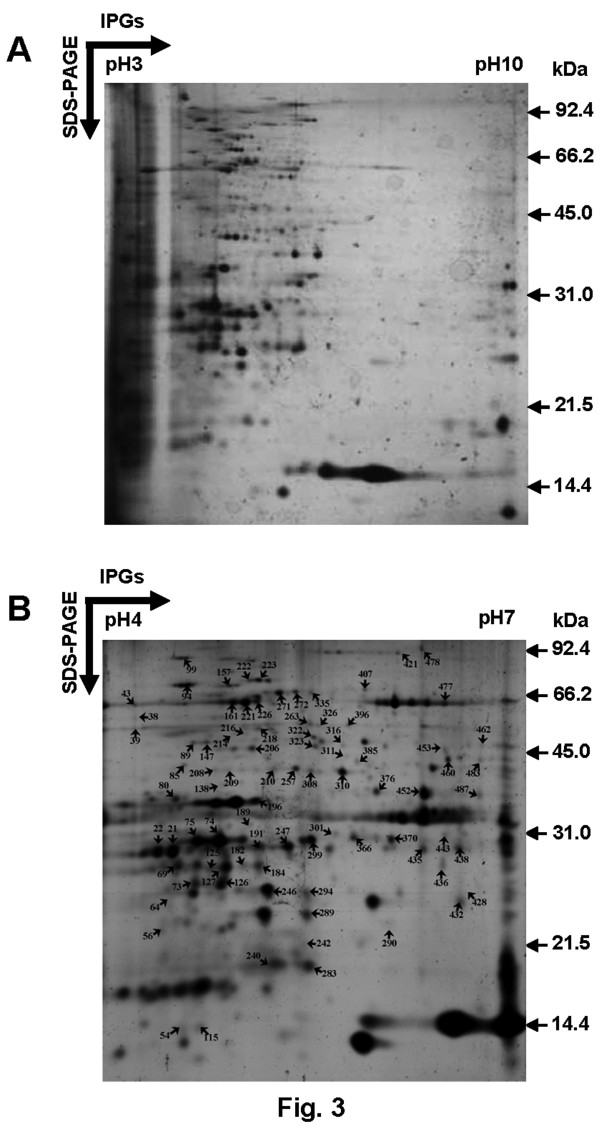
** Chickpea membrane proteome map.** 2-DE was carried out using 100 μg membrane proteins and electrofocused using linear 13 cm IPG strips, pH 3–10 (**A**) and pH 4–7 (**B**). The second dimension was performed using 12.5% SDS-PAGE and protein spots were visualised using MS-compatible silver stain. The identified protein spots were marked by arrows and the numbers corresponding to the spot Id are listed in Table 1. The experiments were performed with at least two biological and three experimental replicates.

To optimize the 2-DE membrane protein separation and resolution, several technical modifications were attempted. Electrofocusing of membrane proteins was performed using different IPG strips (pH 3–10 and 4–7) and various sample-loading methods viz. in-gel rehydration or cup sample loading at the anodic or at the cathodic end of the strips. The proteins were well resolved in cup sample loading at the anodic end with maximum representation of spots in the pH range of 4–7 (Figure 3A and B, Additional file 1. Figure S1). The experiments were performed with at least two biological and three technical replicates. Positional reproducibility in 2-DE was determined by PDQuest analysis that led to the detection of over 300 protein spots of which 280 spots were highly reproducible. The spots were screened with various stringent filtration criteria including spot quality score (>30). MS/MS analysis led to the identification of 91 proteins with high confidence, which is listed in Table 1. The spots are designated as CaM-X, where Ca indicates the organism (*Cicerarietinum*), and M indicates the fraction (membrane) while numerals indicate the spot numbers.

**Table 1:** Proteins identified in membrane fraction using 2-DE coupled with MS/MS analysis

**Functional Category**	**Spot No.**^**a**^	**Identification**	**Score**	**gi No.**^**b**^	**NP**^**c**^	**GRAVY Value**^**d**^	**% Coverage**	**Theoretical MW/pI**	**Experimental MW/pI**
**Signaling and stress response**	CaM-89*	Serine threonine kinase homolog COK-4	48	9796478	2	−0.036	5	42.25/6.42	44.78/4.74
	CaM-246!	Putative serine/threonine kinase	71	110289142	11	−0.526	25	50.40/9.89	25.76/5.30
	CaM-247¶	Membrane protein CH1-like	41	115441379	3	−0.510	2	70.30/4.91	30.04/5.45
	CaM-85!	Calcineurin-like phospho-esterase family protein	63	22329383	11	−0.370	29	43.99/5.36	42.64/4.72
	CaM-436*	Putative quinone oxidoreductase	158	21068664	4	−0.124	25	21.70/6.51	28.26/6.62
	CaM-73*	Actin (Fragment)	63	111610552	1	−0.165	7	23.51/4.78	26.43/4.75
	CaM-428*	Beta-1,3-glucanase	80	116490100	1	0.060	3	36.41/8.82	24.77/6.75
	CaM-182*	Ferritin^e^	50	224140479	1	−0.284	6	16.58/5.14	28.10/5.12
	CaM-487*	Guanine nucleotide-binding protein subunit beta-like	297	3023847	7	−0.153	30	31.83/6.44	36.41/6.89
	CaM-191*	Ferritin^e^	129	255637227	2	−0.297	9	30.24/5.23	30.24/5.23
	CaM-326¶	Cytochrome P450	32	15238866	2	−0.218	4	58.91/7.98	52.62/5.68
	CaM-432¶	Disease resistance protein (Fragment)	32	108739945	2	−0.119	4	23.29/6.10	21.25/6.62
	CaM-94*	Chaperonin 60 alpha^f^ subunit	764	217074850	17	0.004	28	62.02/5.11	72.03/4.7
	CaM-99*	Chaperone DnaK	501	92870233	11	−0.330	15	75.71/5.19	83.09/4.69
	CaM-483*	Luminal binding protein, BiP	248	297742397	7	−0.374	6	73.31/4.96	30.25/6.91
	CaM-157*	Putative GloEL protein Chaperonin 60 kDa	192	225435794	4	−0.094	38	17.51/10.05	45.83/5.00
	CaM-150!	Chaperonin 60 alpha^f^ subunit	108	3790441	12	0.001	13	61.45/5.23	57.09/5.12
	CaM-421¶	Chaperonin 60 alpha^f^ chain precursor,	78	1710807	4	−0.025	7	61.99/5.15	85.92/6.28
**Protein synthesis and degradation**	CaM-39*	Putative 60S ribosomal protein L1	101	84468414	3	−0.445	7	44.93/10.40	51.00/4.29
	CaM-240*	40S ribosomal protein S12	54	255626071	2	0.106	12	15.34/5.50	19.26/5.37
	CaM-283*	40S ribosomal protein S12	162	255626071	3	0.106	23	15.345/5.50	19.02/5.607
	CaM-223!	Putative glutamate-tRNA ligase	70	115443869	14	−0.304	11	63.10/6.27	74.10/5.23
	CaM-208*	Putative 60S acidic ribosomal protein P0	89	84468360	2	0.031	7	34.31/5.27	40.24/5.10
	CaM-209*	Putative 60S acidic ribosomal protein P0	82	84468360	2	0.031	7	34.31/5.27	40.25/5.21
	CaM-301*	Proteasome subunit alpha type	199	224139394	4	−0.227	21	27.38/5.59	31.39/5.81
	CaM-249!	Polyubiquitin	64	18421671	10	−0.281	27	36.40/5.83	28.56/5.47
	CaM-407!	Mitochondrial processing peptidase beta subunit	394	12802327	20	−0.302	22	58.90/6.56	70.46/6.03
**Bioenergy**	CaM-242*	Cytochrome b6-f complex iron-sulfur subunit	76	136707	2	−0.039	15	16.85/6.45	22.19/5.53
	CaM-21!	Chlorophyll a/b-binding^g^ protein AB96	224	115773	9	0.011	22	24.36/4.98	29.51/4.58
	CaM-75*	Chlorophyll a/b binding^g^ protein	411	3928140	12	−0.068	30	28.35/5.47	30.58/4.78
	CaM-196*	Oxygen-evolving enhancer protein 1^h^	177	131384	6	−0.303	15	35.44/5.19	35.44/5.19
	CaM-294*	Putative PSII-P protein	104	217072770	2	−0.273	8	28.41/7.12	25.41/5.59
	CaM-22!	Chlorophyll a/b-binding protein 3 precursor^g^	160	115800	7	0.005	19	27.90/5.46	29.38/4.47
	CaM-69!	Chlorophyll A-B binding protein^g^	118	169124051	8	0.071	12	28.59/5.44	28.05/4.62
	CaM-126!	Chlorophyll a/b-binding protein type III (Fragment)^g^	65	7271947	4	−0.013	9	20.81/5.17	26.40/4.95
	CaM-127!	PSI type III chlorophyll a/b-binding protein (imported)	99	15219941	7	−0.014	11	29.16/8.61	27.98/4.98
	CaM-125!	Oxygen-evolving enhancer protein 1^h^	111	131384	7	−0.303	14	35.10/6.25	27.83/4.87
	CaM-183!	Oxygen-evolving enhancer protein 2	89	131390	9	−0.312	14	28.20/8.29	25.64/5.16
	CaM-376*	Ferredoxin NADP reductase	81	125553745	2	−0.340	5	39.95/8.72	36.9/6.12
	CaM-184¶	LHCA3, chlorophyll binding	58	79320443	4	−0.009	18	23.84/5.62	28.07/5.23
	CaM-74!	Light-harvesting chlorophyll-a/b binding protein Lhcb1	387	56809379	13	−0.083	19	28.43/5.48	30.58/4.93
	CaM-319!	Putative ferredoxin-NADP(H) oxidoreductase	67	41052915	7	−0.383	19	41.09/7.98	39.76/5.92
	CaM-452¶	Ferredoxin: NADP + reductase	130	4930119	3	−0.403	12	34.98/6.54	36.58/6.49
	CaM-226!	ATPase beta subunit (Fragment)^i^	276	6467935	18	−0.026	27	51.53/5.22	65.89/5.21
	CaM-147*	ATP synthase subunit beta, chloroplastic^i^	92	11466372	2	0.007	6	51.99/5.10	44.87/4.84
	CaM-189*	ATP synthase subunit alpha^j^	589	197294119	18	−0.057	24	55.64/5.44	32.38/5.16
	CaM-221!	ATP synthase beta subunit (Fragment)^i^	116	3850926	13	0.001	22	52.73/5.15	65.80/5.14
	CaM-161!	ATP synthase beta subunit (Fragment)^i^	918	7708546	41	0.004	51	51.18/5.07	65.76/5.04
	CaM-80!	ATP synthase beta subunit (Fragment)^i^	671	4063542	19	0.009	26	49.13/5.06	35.75/4.60
	CaM-316*	ATP synthase subunit alpha^j^	328	197294119	10	−0.057	17	55.64/5.44	45.62/5.83
	CaM-222*	ATP synthase subunit beta^i^	410	197294097	19	−0.016	27	52.96/5.16	73.99/5.16
	CaM-335*	ATP synthase subunit alpha^j^	75	197294119	2	−0.057	4	55.64/5.44	69.22/5.62
	CaM-385¶	ATP synthase subunit gamma, chloroplast precursor	77	226533016	1	−0.143	3	40.10/8.44	41.00/6.06
	CaM-311*	ATP synthase CF1 alpha subunit^j^	295	289066833	5	−0.045	11	55.73/5.21	42.49/5.89
	CaM-396*	ATP synthase subunit alpha^j^	439	197294119	10	−0.057	19	55.64/5.44	52.90/5.91
	CaM-272!	ATP synthase CF1 alpha subunit^j^	572	13518443	21	−0.055	23	55.80/5.22	69.51/5.51
	CaM-271!	H + −transporting two-sector ATPase alpha chain	83	114522	13	−0.044	21	54.64/5.75	69.09/5.38
**Metabolism**	CaM-218*	Glutamine synthetase	50	12963877	1	−0.393	2	47.40/5.73	50.72/5.32
	CaM-308*	Fructose-bisphosphate aldolase 1^k^	182	399024	5	−0.172	14	38.63/5.83	39.99/5.62
	CaM-310¶	Fructose-bisphosphate aldolase (EC 4.1. 2.13) precursor^k^	206	399024	6	−0.172	17	38.74/5.83	39.89/5.86
	CaM-453*	Fructose-bisphosphate aldolase^k^	122	3913008	4	−0.148	14	38.42/6.21	37.10/6.70
	CaM-322*	Phosphoglycerate kinase precursor like	124	82621134	5	0.128	11	50.35/8.19	46.94/5.64
	CaM-257¶	Plastidic aldolase^k^	240	38096041	9	−0.151	17	43.18/6.86	35.52/5.61
	CaM-210*	Fructose-bisphosphate aldolase^k^	163	84468290	5	−0.140	14	43.03/6.86	40.35/5.35
	CaM-460!	Glyceraldehyde 3-phosphate dehydrogenase^l^	70	3413165	4	0.069	15	20.88/5.24	41.91/6.66
	CaM-216¶	Glutamate-ammonia ligase (EC 6.3.1.2) delta precursor	39	121344	1	−0.390	2	47.65/6.18	51.86/5.13
	CaM-115!	Trypsin inhibitor, chain B (fragments)	144	3318877	7	−0.281	57	19.29/4.79	14.86/5.07
	CaM-214¶	Glutamate-ammonia ligase (EC 6.3.1.2) delta precursor	68	121344	1	−0.390	2	47.65/6.18	51.14/5.23
	CaM-366*	Carbonic anhydrase^m^	55	20502881	2	−0.159	4	35.28/6.96	31.01/5.95
	CaM-370*	Carbonic anhydrase^m^	67	20502881	2	−0.159	5	35.28/6.96	30.85/6.23
	CaM-206!	Phosphoribulokinase	71	1885326	6	−0.289	7	39.23/5.41	43.82/5.18
	CaM-435*	Carbonic anhydrase^m^	95	8954289	3	−0.091	6	35.46/7.59	29.73/6.47
	CaM-438*	Carbonic anhydrase^m^	56	8954289	2	−0.091	4	35.46/7.59	29.78/6.74
	CaM-462*	Glyceraldehyde-3-phosphate dehydrogenase A^l^	200	120658	6	−0.047	12	43.31/8.80	44.49/6.81
**Miscellaneous**	CaM-155!	Dynein-1-alpha heavy chain	69	159490411	63	−0.257	10	525.42/5.32	61.98/5.11
	CaM-54!	Activator of spomin	65	145357734	8	−0.634	24	17.25/9.63	12.38/4.69
	CaM-289¶	Ribulose-1,5-biphosphate carboxylase/oxygenase large subunit	129	10764577	5	−0.254	10	51.58/5.96	23.69/5.59
	CaM-299!	Histone acetyltransferase GCN5	79	18410098	15	−0.581	26	63.48/6.01	30.69/5.63
	CaM-38*	Ribulose bisphosphate carboxylase/Oxygenase large chain	60	1750362	2	−0.249	4	52.22/6.09	56.17/4.30
	CaM-138*	Chromosome chr17 scaffold_16	149	225456471	4	−0.342	4	33.35/5.67	37.90/4.97
	CaM-43*	Ribulose bisphosphate carboxylase large chain	295	197294096	8	−0.277	16	52.65/6.04	64.35/4.29
	CaM-290!	Intronic ORF, similar to LAGLIDADG endonuclease	80	2943730	11	−0.486	21	29.46/10.01	21.47/6.00
**Unknown function**	CaM-443*	Predicted protein	53	224119706	1	0.038	3	27.44/8.65	31.20/6.64
	CaM-263¶	OSJNBb0085H11.1 protein	50	38346013	1	-.293	7	176.76/9.26	53.68/5.61
	CaM-478*	Putative uncharacterized protein	64	147828109	19	−0.788	0	58.79/6.46	87.91/6.46
	CaM-323*	Putative uncharacterized protein	94	224284512	2	−0.395	7	40.97/6.04	44.21/5.66
	CaM-477!	Unknown protein	67	27497205	16	−1.250	8	16.57/10.81	65.11/6.62
	CaM-171!	Unknown protein	67	15236812	16	−0.827	8	90.90/5.09	74.93/5.11
	CaM-64!	Hypothetical protein P0415D04.53	66	47496995	8	−0.587	34	30.31/11.81	24.12/4.77
	CaM-267!	Hypothetical protein F26P21.180	67	3688187	10	−0.645	22	57.25/7.64	44.54/5.58
	CaM-56!	Hypothetical protein OSJNBa0075N02.148	66	28972006	7	−1.039	29	20.62/12.24	22.37/4.49

^a^Spot number as marked on the proteome (Figure 3B). The spot numbers are designated as CaM-X, where Ca indicates the organism (*Cicerarietinum*), M denotes the fraction (membrane), and X corresponds to the spot number. ^b^Protein identification number as in GenBank. ^c^NP represents the number of peptides. ^d^GRAVY value calculated using ProtParam tool available on ExPasy server. ^e-m^ denotes the respective protein(s) identified from multiple spots. This could be caused by posttranslational modification(s) or different isoforms, protein degradation/synthesis. Spots marked by ‘*’ were identified by Q-TRAP, ‘¶’ by QStar, and ‘!’ by MALDI TOF-TOF analysis.

### Physicochemical characteristics of membrane proteins

**Figure 4:**
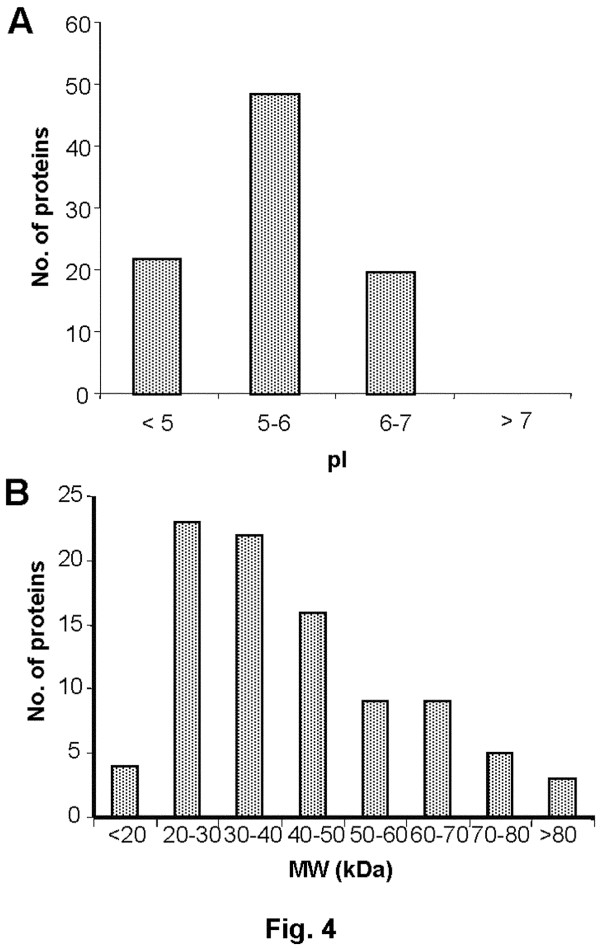
** Characteristic features of membrane proteins of chickpea.** Distribution of the identified proteins in relation to their pI (**A**) and molecular mass (**B**) are shown. The proteins were sorted based on their experimental molecular mass and pI ranges as indicated.

To examine putative characteristic features of the proteome, the identified proteins were analysed with respect to molecular mass and pI distribution. The pI of the proteins identified in chickpea ranges from 4 to 7 (Figure 4A). This distribution is not radically different from that of *Medicago* with 85% proteins distributed in similar pI range [20]. However, a comparison with that of white lupin indicated slight differences with 55% basic proteins (pI > 7) [21]. The molecular masses of proteins identified ranged from 10 to 90 kDa, with majority of proteins (~80%) exhibiting a molecular mass between 20 and 60 kDa (Figure 4B). This seems to be a relatively classical feature of legume membrane proteins. 

**Figure 5:**
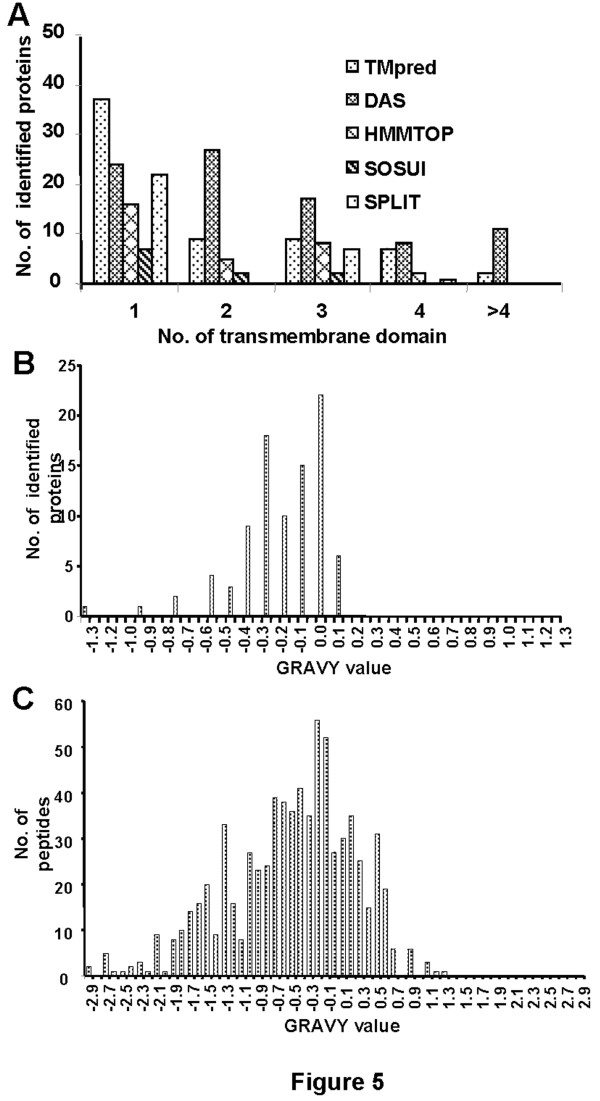
** Physicochemical characteristics of the identified membrane proteins.** The predicted topology of the identified proteins in terms of TMDs, determined by TMpred, DAS, HMMTOP, SOSUI and SPLIT is shown in (**A**). The hydropathy profile (GRAVY index) of the identified proteins (**B**) and that of peptides (**C**) are shown. GRAVY scores of the identified proteins and peptides were rounded to one decimal place and plotted.

To evaluate the physicochemical characteristics of the chickpea membrane proteins, the whole dataset was analysed in terms of hydrophobicity (grand average of hydropathicity, GRAVY values) and membrane localization vis-à-vis the presence of TMDs. The modular structure of membrane proteins consist of hydrophobic domains and hydrophilic loops or termini. The hydrophobic regions are involved in the formation of TMDs that may be α-helices or β-barrels, deeply buried in the lipid bilayer [22]. Five different prediction programs were used to predict putative TMDs in the identified proteins ( Additional file 2. Table S1). Figure 5A shows the distribution of identified proteins with one or multiple membrane-spanning domains. Significantly, 70% of the identified proteins are designated as having putative TMDs, based upon TMpred analysis. It is notable that the number of integral membrane proteins identified in this study is significantly higher than those reported in previous gel-based membrane proteome analyses (Table 2). 

**Table 2:** Integral membrane proteins identified in membrane proteomes

**Plant**	**Total number of identified proteins**	**Integral proteins (having putative TMD**^**a**^**)**	**% of integral proteins identified**
*Cicer arietinum*	91	64	70.3
*Medicago truncatula*^*b*^	96	22	22.9
*Lupinus albus*^*c*^	37	18	48.6

^a^TMpred used as prediction tool. ^b^Calculations adapted from TMpred analysis [6]. ^c^Additional file 3. Table S2 contains details of TMpred analysis as applied to the membrane proteome dataset of white lupin.

Another valid indicator of the membrane association of proteins is their GRAVY value in the positive range, depicting their hydrophobic nature [23,24]. GRAVY value analysis was performed at the protein (Table 1) as well as peptide level ( Additional file 4. Table S3). The positive GRAVY value of the identified proteins ranged from 0.001 to 0.128 (Figure 5B), and that of the peptides from 0.014 to 1.287 (Figure 5C). While some proteins such as membrane protein CH1-like (CaM-247) with three putative TMDs showed negative GRAVY value (−0.510), glyceraldehyde 3-phosphate dehydrogenase (GAPDH, CaM-460) with no predicted TMD scored positive GRAVY value (0.069). It has been reported that most of the integral cytoplasmic membrane proteins are hydrophobic, while the majority of integral outer membrane proteins are hydrophilic [4,24], causing the observed ambiguity in GRAVY values.

### Analysis of functional groups of membrane proteins

**Figure 6:**
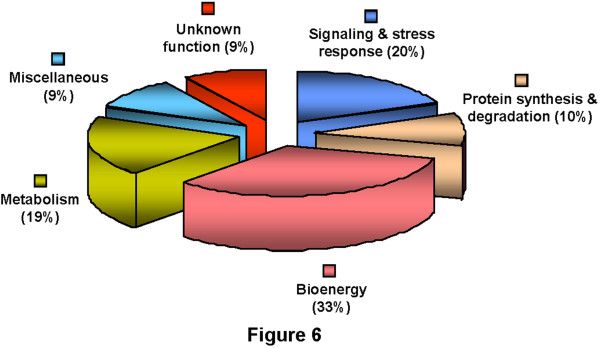
** Functional classification of the membrane proteins of chickpea.** The identified proteins were catalogued based upon their putative functions, assigned using protein function databases and literature search.

The identified proteins were functionally classified into various categories (Figure 6) based on protein function database Pfam or InterPro and literature search. The assigned classes include bioenergy (33%), signaling and stress response (20%), metabolism (19%), protein synthesis and degradation (10%), miscellaneous (9%) and proteins with unknown function (9%). In a number of cases, the same protein was identified from multiple spots in the same gel. This suggests the possible posttranslational modification(s), which might lead to the change in isoelectric point and or molecular weight. For example, spots CaM-21 and 69 were identified as chlorophyll a/b-binding protein but they showed different pIs and molecular weights.

We identified many candidates that are known membrane residents, and also membrane-associated proteins. The most abundant class of proteins belonged to ‘Bioenergy’. Members in this class are mainly associated with energy-production processes such as photosynthesis and oxidative phosphorylation. In green plants, chloroplast and mitochondria are the most prominent and abundant organelles. Most of the identified proteins in ‘Bioenergy’ belong to these organelles, such as the proteins involved in the formation of major complexes of photosynthetic apparatus such as light harvesting antenna complexes associated with PSI and PSII (CaM-21, 22, 69, 74 126, 127, and 184), cytochrome b6-f complex (CaM-242) and oxygen-evolving enhancer protein 1 and 2 (CaM-125 and 183). Protein spots CaM-319, 376 and 452 were identified as ferredoxin NADP reductase (FNR), which is tightly bound to the thylakoid membrane [25] and cause the reduction of NADP^+^ during photosynthetic electron transport. Multiple subunits of ATP synthase such as alpha, beta and gamma were also identified.

The class ‘Metabolism’ contained proteins like glutamine synthetase (CaM-218) and glutamate ammonia ligase delta precursor (CaM-214 and 216), which are primarily involved in nitrogen metabolism. The protein spot CaM-322 was identified as phosphoglycerate kinase (PGK), which has been found to be associated with the thylakoid membrane in higher plants [26]. Carbonic anhydrase, known to provide inorganic carbon for improved photosynthetic efficiency, was identified from multiple spots (CaM-366, 370, 435, and 438). Although carbonic anhydrase is primarily located in the chloroplast, it is also reported to be present in the microsomal and plasma membrane fractions [27]. It is increasingly apparent that many enzymes are not free in solution, but interact with membrane structures [28] or with other proteins [29]. The presence of metabolic pathway enzymes like aldolase as identified from multiple spots (CaM-210, 257, 308, 310, and 453), phosphofructokinase (CaM-206), and GAPDH (CaM-460 and 462), among others, highlights this phenomenon. It has been reported that aldolase interacts with V-ATPase [30], which may provide a basis for coupling glycolysis directly to the ATP-hydrolyzing proton pump [31]. In *Arabidopsis*, 5-10% of the cytosolic isoforms of each glycolytic enzyme is associated with the outer surface of mitochondria [32]. These glycolytic enzymes associated with mitochondria are catalytically competent and constitute a functional glycolytic pathway [33], though the significance of this micro-compartmentation of glycolysis has not been fully understood.

A subset of the identified proteins was presumably involved in ‘Signaling and stress response’. This class included proteins such as calcineurin-like phosphoesterase (CaM-85) and membrane protein CH1-like (CaM-247), the latter having putative SUN (Sad1/UNC-84) domain. Proteins having the SUN domain are involved in the formation of bridging structures, LINC (linker of nucleoskeleton and cytoskeleton) complex that plays an important role in DNA duplication, especially in the anchorage of centrosomes and spindle pole body to the nuclear envelope [34,35]. The discovery of SUN proteins in plant established the existence of LINC complex [36]. In animal, SUN proteins are known to involve in the transfer of mechanical force generated in cytosol to inner nuclear membrane [37], but such role in plants is unknown. Calcineurin-likephosphoesterase belongs to the protein family that includes diverse range of phosphoesterases, phosphoserine phosphatases, and nucleotidases. The exact role of this protein in signaling is not known; however, it is likely to be involved in phosphate mobilization either by cleavage of phosphate from phosphate-containing compounds or by metabolic changes [38]. The spots CaM-89 and 246 were identified as serine/threonine kinase. These kinases may act as receptor, which interact with other proteins to affect a wide array of processes, specifically in stress adaptation [39]. CaM-436 was identified as quinone oxidoreductase, a class of membrane enzymes that catalyse the oxidation or reduction of membrane-bound quinols/quinones. This protein has been reported to be associated with the plasma membrane [40] and microsomal membranes [41]. Protein spot CaM-326 was identified as cytochrome P450, one of the largest super families of enzymes. It uses electrons from NAD(P)H to catalyze activation of molecular oxygen, leading to regiospecific and stereospecific oxidative attack of a plethora of substrates [42,43]. Plant P450s are a class of proteins anchored on the cytoplasmic side to ER [44], but is also found in inner mitochondrial membranes [45] as well as in tonoplasts [46].

In this class, another interesting candidate (CaM-89) is the one encoded by COK-4 gene that confers resistance to anthracnose caused by the fungal pathogen *Colletotrichum.* In addition to displaying high similarity to the *Pto* kinase, one of the best characterized *R* gene products, the predicted COK-4 protein contains a highly hydrophobic membrane-spanning region [47]. CaM-432 was also depicted to be a disease resistance protein containing LRR repeat. CaM-483 was identified as BiP, the ER-resident molecular chaperone involved in ER stress signaling. In plants, BiP plays a key role in attenuating ER stress and suppresses the activation of the unfolded protein response [48]. We also identified HSPs such as chaperone DnaK (CaM-99), chaperonin-60 (CaM-94, 150, and 421), and GloEL protein (CaM-157), which prevent protein misfolding and random aggregation inside the cell. The major HSP families are necessary for the assembly and unfolding or transport of proteins through membranes [49,50].

The class ‘Protein synthesis and degradation’ predominantly include ribosomal proteins (CaM-39, 208, 240, 208, 209 and 283), putative glutamate tRNA ligase (CaM-223), proteasome subunit alpha type (CaM-301), polyubiquitin (CaM-249), and mitochondrial processing peptidase beta subunit (CaM-407). It has been reported that polysomes are linked to actin filaments, which in turn are associated with the plasma membrane [51]. Furthermore, ribosomes are also attached to the ER and nuclear membrane through the larger subunit. The most common pathway for degradation of cellular proteins is the ubiquitin proteasome pathway. We could identify proteins associated with these pathways such as polyubiquitin (CaM-249) and proteasome subunit alpha type (CaM-301). Ubiquitin is known to be the most conserved protein required for ATP-dependent protein degradation and involved in protein transport. These proteins were identified from the tonoplast [30], as well as from the plasma membrane [52].

The ‘Miscellaneous’ class accounted for 9% of the proteins identified. This class include activator of spomin (CaM-54), dynein-1-alpha heavy chain (CaM-155), chromosome chr17 scaffold_16 (CaM-138), and histone acetyltransferase (HAT, CaM-299), among others. HAT has been linked to transcriptional activation of various genes and is known to associate with mammalian inner nuclear membrane [53]. The activator of spomin is known to involve in the activation of a subset of sugar-responsive genes and control the carbon flow in plants [54]. Chromosome chr17 scaffold_16 encodes for ATP-dependent Clp protease ATP-binding subunit. Clp proteases are known to degrade both soluble and membrane-bound substrates [55] and are reported to be associated with the stroma as well as the inner envelope membrane in plants [56]. The proteome map revealed 9% proteins as hypothetical or proteins with unknown function, which were subjected to domain analysis using InterProScan. The analysis led to the identification of different conserved domains, thereby providing valuable insight into their functional implications ( Additional file 5. Table S4). The putative conserved domain was bacterial transferase hexapeptide repeat in CaM-443, kinesin-related domain in CaM-56, and ATPase, AAA type core in CaM-323, among others. Intriguingly, membrane receptors, aquaporins, and transporters could not be identified, possibly due to their low abundance and complex physicochemical properties. Nevertheless, the distribution of various functional classes bear resemblance to previously reported membrane proteome datasets of rice [7].

### Comparative analysis of membrane proteomes

**Figure 7:**
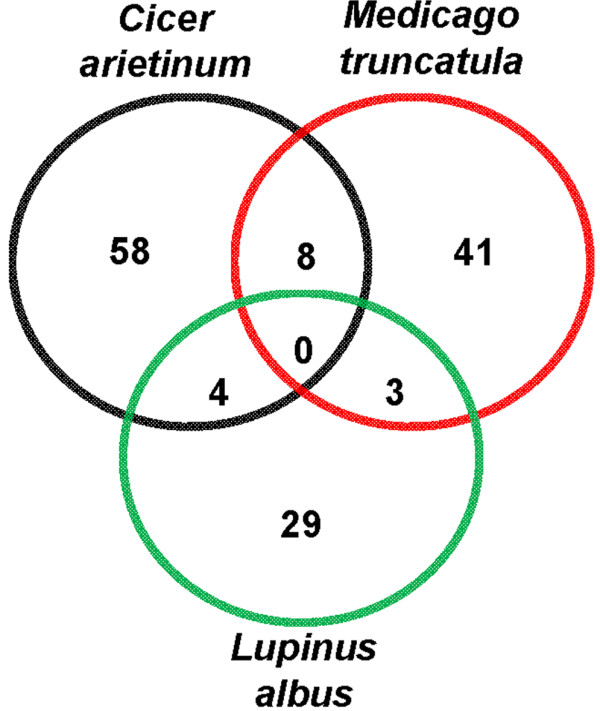
** Comparative Analysis of Plant Membrane Proteomes**. Venn diagram showing the distribution of common and exclusive proteins amongst *C*. *arietinum*, *M. truncatula* and *L. albus*. The areas shown in the diagram are not proportional to the number of proteins in each group.

To investigate the comparative proteomics of membrane proteins at organismal level, the representative membrane proteomes of *Medicago truncatula*[20] and *Lupinus albus*[21] were compared with that of chickpea. For the purpose, we considered only gel-based proteomes of the microsomal fraction. Very few proteins were found to be common between any two datasets and interestingly, none of the proteins was found to be common amongst all the datasets (Figure 7, Additional file 6. Table S5). The percentage of proteins found to be exclusive to each proteome varied as 83% in chickpea, 79% in *Medicago* and 80% in white lupin. Given the comparison between the proteome of any two species, chickpea showed higher percentage of similarity with *Medicago*. The poor overlap amongst the proteome datasets can be attributed to the fact that the representative proteomes are unsaturated and more so, the microsomal fraction of chickpea was extracted from aerial tissues as against root tissues used for *Medicago* and white lupin. 

## Conclusions

Most plant membrane proteomes till date have been developed from roots, the notable exceptions being leaves [57], trichomes [58], seedlings [59], and aerial tissues (this study). Unlike root tissues that arise from root apical meristems, the aerial tissues are initiated by the shoot apical meristems, which show more diversity. In recent years, shoot meristems have received considerable attention in view of their importance in plant development and stress adaptation [60]. Further, only a small fraction of integral membrane proteins have been confirmed experimentally, and most of them came from *in silico* analyses of genome datasets of the model plants. The difficulties associated with the study of membrane proteins in non-model plants has been demonstrated in a recent proteomic study [61]. This underscores the importance of the study of membrane proteins in chickpea, whose genome is yet to be sequenced. This study provides a firm indication of a number of membrane-associated proteins to which function is yet to be assigned. The proteome revealed many key membrane-associated proteins, for example, serine threonine kinase homolog COK4, calcineurine-like phosphoesterase, activator of spomin, and chromosome chr17 scaffold_16, among others, which were not reported earlier.

## Methods

### Plant materials and growth condition

Chickpea (*Cicer arietinum* L.) seeds were soaked in water, kept overnight in dark and grown in pots (10 seedlings/1.5 L capacity pots with 18 cm diameter) containing a mixture of soil and soilrite (2:1, w/w). The seedlings were maintained at 25 ± 2°C, 50 ± 5% relative humidity under 16 h photoperiod (270 μmol m^-2^ s^-1^ light intensity) as described previously [17]. The pots were provided with 100 ml of water every day that maintained the soil moisture content to approx. 30%. The aerial parts (stem and leaves) of 3-week-old seedlings were sampled as experimental materials.

### Isolation of membrane fraction

The membrane i.e., microsomal fraction was isolated as described earlier [62] with few modifications. Approximately, 10 g tissue was ground into powder in liquid nitrogen with 1% (w/w) polyvinylpolypyrroledone (PVPP). The tissue powder was homogenized in homogenizing buffer [0.25 mM sucrose, 3 mM EDTA, 5 mM DTT, 10 mM ascorbic acid, 70 mM Tris-MES (pH 8.0), 0.25 mM PMSF and protease inhibitor cocktail (Sigma)] using a homogenizer (PRO Scientific, USA). The cell debris was removed from the homogenate by filtration through four-layered cheese cloth and the filtrate was centrifuged at 6000 × *g* for 10 min at 4°C. The supernatant was recovered and centrifuged at 150000 × *g* for 45 min at 4°C. The resulting pellet containing the membrane fraction was suspended in suspension buffer (1.1 mM glycerol, 5 mM DTT, and 10 mM Tris-MES, pH 8.0).

### Marker enzyme assays

The presence of different subcellular membranes was determined by assaying activity their respective marker enzymes. The activities of orthovanadate-, azide-, nitrate-sensitive ATPase and latent IDPase were measured for plasma membrane, mitochondrial membrane, tonoplast and Golgi membrane, respectively [63,64]. In brief, 30 μg membrane proteins were suspended in 30 mM Tris-MES [pH 6.5 (vanadate-sensitive ATPase), pH 8.0 (nitrate and azide-sensitive ATPase), and pH 7.5 (latent IDPase)]. The reactions were performed in 1 ml solution containing 3 mM MgSO_4_, 0.1 mM sodium molybdate, 50 mM KCl and 3 mM ATP, with or without ATPase inhibitor (100 μM Na_3_VO_4_, 50 mM KNO_3,_ and 1 mM NaN_3_) at 38°C to minimise the hydrolysis of ATP. To subtract the residual Pi in isolated fraction, the reactions were also performed without addition of ATP. The released Pi was determined as described previously [65]. The reaction was stopped by the addition of 1 ml Ames’s colour reagent [1 part ascorbic acid (10%) and 6 parts ammonium molybdate (0.42% in 1 N H_2_SO_4_)] containing 0.1% (w/v) SDS. The colour was allowed to develop for 20 min. After termination with 10% (w/v) sodium citrate, the absorbance was measured at 820 nm. For latent IDPase, the reaction was carried out with 3 mM IDP-salt, either in presence or absence of 0.1 mM sodium molybdate. The activity was determined on freshly isolated membrane fraction then after 5 days of incubation at 4°C.

### Extraction and quantification of membrane proteins

Membrane proteins were extracted using the organic solvent mixture of chloroform/methanol as described previously [66]. The microsomal fraction was suspended in 1 ml of suspension buffer and divided in 10 sub-fractions of 0.1 ml. The aliquots were slowly added to 0.9 ml of cold chloroform/methanol mixtures (0:9 to 9:0, v/v) and kept on ice for 15 min. Intermittent vortexing of samples was carried out during the incubation. The mixtures were centrifuged at 12000 × g for 20 min at 4°C. The pellet fractions containing insoluble proteins were retrieved carefully and to the respective supernatants, 2–5 volumes of chilled acetone was added, followed by incubation at −20°C and the precipitated proteins collected by centrifugation. The soluble and insoluble fractions’ membrane proteins were resuspended in the buffer containing 7 M urea, 2 M thiourea, and 4% CHAPS (w/v). The protein concentration was determined using the 2-D Quant kit (GE Healthcare).

### Measurement of phytopigments

The spectrophotometric assay was used to determine the content of phytopigments such as chlorophylls and carotenoids. After removal of insoluble proteins, equal volume of organic phase of each ratio of chloroform/methanol was added to 80% chilled acetone. Mixtures were centrifuged at 2700 × g for 10 min and the supernatant was taken to measure the absorbance at 663, 645 and 470 nm, respectively [67]. The amounts of phytopigments were calculated as follows:

(1) $$\begin{array}{c} \text{Chl a}=\left[\left(12.{7\;\text{x A}}_{663}\right)–\left(2.{69\;\text{x A}}_{645}\right)\right],\text{Chl b}=\left[\left(22.{9\;\text{x A}}_{645}\right)–\left(4.{68\;\text{x A}}_{663}\right)\right]\text{ and}\\ \text{ carotenoids}=\left[\left(1000{\;\text{x A}}_{470}\right)–\left(3.27\;\text{x Chla}+1.04\;\text{x Chlb}\right)\right]/227 \end{array}$$

### 2-DE of membrane proteins and data analysis

Since the organic solvent extraction at 6:3 ratio of chloroform/methanol was found to be optimal for membrane proteins, isoelectric focusing (IEF) was carried out with 100 μg of proteins from the pellet fraction. The immobilized gel strips (13 cm, pH 3–10 or 4–7, GE Healthcare) were rehydrated overnight in 250 μl of rehydration buffer [7 M urea, 2 M thiourea, 4% (w/v) CHAPS, 60 mM DTT, 2% (v/v) pharmalyte (pH 3–10 or 4–7), and 0.05% (w/v) bromophenol blue]. During cup-loading, proteins were diluted with rehydration buffer to final concentration of 1 μg/μl and loaded either at anodic or cathodic ends. Initially, proteins were electrofocused at lower voltage and later with higher voltage up to 85000 VhT at 20°C using IPGphor system (GE Healthcare). After IEF, the strips were subjected to reduction with 1% (w/v) DTT in 10 ml of equilibration buffer [6 M urea, 50 mM Tris–HCl (pH 8.8), 30% (v/v) glycerol and 2% (w/v) SDS], followed by alkylation with 2.5% (w/v) iodoacetamide in the same buffer. The strips were then loaded on top of 12.5% polyacrylamide gels for SDS-PAGE. The electrophoresed proteins were stained with silver stain plus kit (Bio-Rad). The silver stained gels were scanned with Bio-Rad FluorS equipped with a 12-bit camera. The 2-DE gels were analyzed with PDQuest version 7.2.0 (Bio-Rad). Three replicate 2-DE gels, corresponding to at least two biological replicates were matched together to generate a composite image conventionally known as first level matchset. Protein spots present in at least two of the three gels were considered for analysis. Experimental molecular mass and pI for each protein were determined from 2-DE image using standard molecular mass protein marker (Bio-Rad).

### Protein identification using MALDI-TOF/TOF and LC-ESI-MS/MS

The protein spots were excised manually, washed twice with deionized water, and trypsin in-gel digestion was performed [17]. The peptide extract was vacuum dried. While reconstitution of the peptides for MALDI-TOF/TOF was performed in 3 μl of 50% (v/v) ACN and 0.1% (v/v) TFA, the same was performed in 7 μl of 50% (v/v) ACN and 0.1% (v/v) HCOOH for LC-MS/MS. Instruments used for analysis were 4800 MALDI TOF/TOF Analyzer (Applied Biosystems), QStar Elite coupled to Tempo nano MDLC (Applied Biosystems) equipped with ion spray source running Analyst QS software, and ultimate 3000 nano HPLC system (Dionex) coupled to a 4000 QTRAP mass spectrometer (Applied Biosystems).

The acquired mass spectra were searched using Mascot search engine (http://www.matrixscience.com). The following parameters were used: maximum allowed missed cleavage 1, fixed amino acid modification as carbamidomethyl and variable amino acid modifications either oxidation (M) or acetyl (N-term) or both, taxonomy set to Viridiplantae/*Oryza*/*Arabidopsis*, and databases used MSDB or Ludwig NR. The peptide and fragment mass tolerance for spectra obtained from MALDI and QStar were 100 ppm, 0.3 Da, and 100 ppm, 0.4 Da, respectively, while for TRAP it was 1.2 and 0.6 Da. Only those protein samples whose MOWSE score [68,69] was above the significant threshold level (p < 0.05) as determined by MASCOT were considered. Since chickpea genome is not sequenced, therefore a homology based search was performed. The details regarding the precursor ion mass, expected molecular weight, theoretical molecular weight, delta, score, rank, charge, number of missed cleavages, peptide sequence, database, taxonomy, and spectra for proteins identified with a single peptide are given in in Additional file 7. A list of each peptide score and threshold score of identified proteins is given in Additional file 8.

### Bioinformatic analysis

The prediction of transmembrane domain (TMD) of the identified proteins were carried out using TMpred (http://www.ch.embnet.org/software/TMPRED_form.html), DAS (http://www.sbc.su.se/~miklos/DAS/), HMMTOP (http://www.enzim.hu/hmmtop/), Sosui (http://bp.nuap.nagoya-u.ac.jp/sosui/sosui_submit.html) and SPLIT (http://split.pmfst.hr/ split/4/). Grand Average of Hydropathicity (GRAVY) value for each protein and peptide was calculated using ProtParam tool available at the Expasy server (http://au.expasy.org/tools/protparam.html). To determine the function of unknown proteins, domain analysis was performed to predict the conserved domain using the InterPro (http://www.ebi.ac.uk/interpro/) database and queried for domains in the SMART (http://smart.embl-heidelberg.de/), Panther (http://www.Pantherdb.org/), and Pfam (http://www.sanger.ac.uk/software/Pfam/) databases.

## Abbreviations

2-DE: Two-Dimensional gel Electrophoresis; DTT: Dithiothreitol; GRAVY: Grand Average of hydropathicity; IDPase: Inosine Diphosphatase; IEF: IsoElectric Focusing; TMD: TransMembrane Domain; SDS: Sodium Dodecyl Sulphate; PAGE: Polyacrylamide Gel Electrophoresis.

## Competing interests

The authors declare that they have no competing interests.

## Author’s contributions

DKJ, AD, SC and NC designed the study, DKJ, PS, and DR carried out the experiments, PM and SG performed the bioinformatic analysis, DKJ, DR, SC and NC wrote the manuscript. All authors read and approved the final manuscript.

## Supplementary Material

Additional file 1 ** Figure S1.** 2-DE gel profile of chickpea membrane proteins using different sample loading methods and variable range IPG strips. Click here for file

Additional file 2 ** Table S1.** Prediction of TMDs in the identified proteins using five different programs. Click here for file

Additional file 3 ** Table S2.** Prediction of TMDs (based upon TMpred) in the membrane proteome dataset of Lupinus albus. Click here for file

Additional file 4 ** Table S3.** Calculation of GRAVY value of each peptide identified by MS/MS analysis. Click here for file

Additional file 5 ** Table S4.** Domain analysis of proteins of unknown function. Click here for file

Additional file 6 ** Table S5.** Comparative analysis of membrane proteome datasets of different plants. Click here for file

Additional file 7 Details of mass spectra of proteins identified with single peptide. Click here for file

Additional file 8 Details of each peptide score and threshold score of identified proteins. Click here for file
