# Supplementary figures and images for: Proteomic analysis reveals the diversity and complexity of membrane proteins in chickpea (Cicer arietinum L.)
Source: Proteome Sci. 2012 Oct 2;10:59. doi: 10.1186/1477-5956-10-59 (PMC3558352; doi:10.1186/1477-5956-10-59)

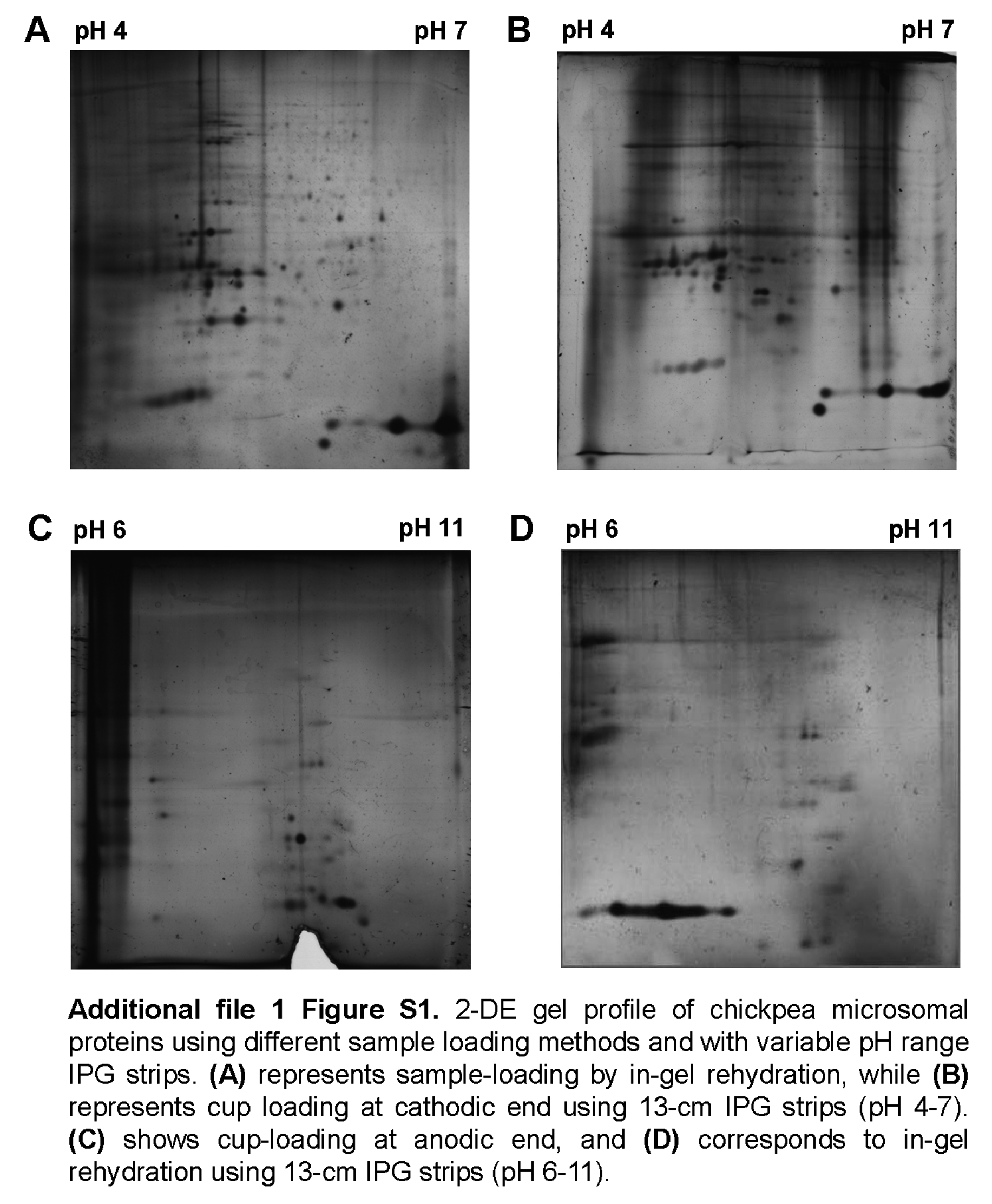

Supplement: Additional file 1 — Figure S1. 2-DE gel profile of chickpea membrane proteins using different sample loading methods and variable range IPG strips. [file 1477-5956-10-59-S1.tiff]
