# Supplementary material for: Proteomic analysis reveals the diversity and complexity of membrane proteins in chickpea (Cicer arietinum L.)
Source: Proteome Sci. 2012 Oct 2;10:59. doi: 10.1186/1477-5956-10-59 (PMC3558352; doi:10.1186/1477-5956-10-59)
Supplement: Additional file 5 — Table S4. Domain analysis of proteins of unknown function. [file 1477-5956-10-59-S5.doc]

**Additional file 4 Table S3 Domain analysis of proteins with unknown function**.

| **Spot No.^a^** | **Identification** | **gi No.^b^** | **InterPro ID^c^** | **Identified Domain** |
| --- | --- | --- | --- | --- |
| CaM-477 | Unknown protein | 27497205 | N/F | N/A |
| CaM-443 | Predicted protein | 224119706 | IPR001451 | Bacterial transferase hexapeptide repeat |
| CaM-263 | OSJNBb0085H11.1 protein | 38346013 | IPR000477 | RNA-directed DNA polymerase |
| CaM-478 | Putative uncharacterized  protein | 147828109 | IPR001878 | Zn finger, CCHC type |
| CaM-323 | Putative uncharacterized protein | 224284512 | IPR003959 | ATPase, AAA type core |
| CaM-171 | Unknown protein | 15236812 | IPR010544 | Kinesin-related |
| CaM-64 | Hypothetical protein P0415D04.53 | 47496995 | N/F | N/A |
| CaM-267 | hypothetical protein F26P21.180 | 3688187 | IPR002130 | Peptidyl-prolyl cis-trans isomerase , cyclophilin type |
| CaM-56 | Hypothetical protein OSJNBa0075N02.148 | 28972006 | IPR007649 | Protein of unknown function DUF591 |

^a^ Spot numbers as displayed on the microsomal proteome.

^b^ GenBank accession number of the identified proteins.

^c^ InterPro domain identification number.

^d^ N/F, not found; N/A, not available
